# Anti-proliferative and apoptotic effects of *Ziziphus Jujube* on cervical and breast cancer cells

**Published:** 2016

**Authors:** Mohammad Reza Abedini, Nafiseh Erfanian, Habibollah Nazem, Sara Jamali, Reyhane Hoshyar

**Affiliations:** 1 *Cellular & Molecular Research Center, Birjand University of Medical Sciences, Birjand, Iran *; 2 *Department of Pharmacology, Faculty of Medicine, Birjand University of Medical Sciences, Birjand, Iran*; 3 *Department of Biology, Payam-e Noor University of Esfahan, Esfahan, Iran*; 4 *Department of Biochemistry, Faculty of Medicine, Birjand University of Medical Sciences, Birjand, Iran*

**Keywords:** *Ziziphus Jujube*, *Cervical cancer*, *Breast cancer*, *Apoptosis*

## Abstract

**Objective:**

*Ziziphus Jujube* (Jujube) plant has exhibited numerous medicinal and pharmacological properties including antioxidant and anti-inflammatory effects. This study was carried out to investigate its anti-cancer and pro-apoptotic abilities in human cervical and breast cancer cells in vitro.

**Materials and Methods:**

The cervical OV2008 and breast MCF-7 cancer cells were incubated with different concentrations of Jujube aqueous extraction (0-3 mg/ml) for various times (0-72 h). Cell viability was assessed by Trypan Blue and 3-(4,5-dimethylthiazol-2-yl)-2,5-diphenyltetrazolium bromide (MTT) assay. The expression of two apoptosis-related genes in treated cells evaluated by quantitative Real Time -PCR analysis.

**Results:**

Jujube significantly inhibited cancer cell viability in a dose- and time- dependent manner. Herb-induced apoptosis was associated with enhanced expression of Bax and decreased Bcl2 gene leading eventually to a time-dependent six fold increase in the Bax/Bcl-2 ratio.

**Conclusion:**

These results indicated that Jujube may be a natural potential and promising agent to prevent or treat human cancers.

## Introduction

Cancer is a heterogeneous devastating disease with various biological characteristics (Senapathy et al., 2011 ▶). Breast and cervical cancers are the first and second most common malignancies among women worldwide, respectively (World health organization, 2013). Current therapeutic strategies for cancer such as surgery, radiotherapy and chemotherapy are associated with serious side effects, residual morbidity as well as frequent relapses (Adhvaryu et al., 2008 ▶). A growing body of evidence suggested a promising potential for medicinal plants used as traditional and/or alternative modern medicine. Specifically, there is a considerable interest among oncologists to develop anticancer agents from herbs (Mishra et al., 2011 ▶; Sadiq et al., 2008 ▶; Hoshyar et al., 2015 ▶). Current experiments showed that herbs play anticancer role via induction of program cell death (apoptosis) and cell differentiation, enhancing the immune system potential, inhibiting angiogenesis and reversing multidrug resistance (Liu et al., 2013 ▶). However,mucheffort yet is required to determine the role of herbs in cancer therapy. One of such medicinal plants is Jujube with numerous biological compounds and a long history of use as a remedy for various disorders (Preeti and Tripathi, 2014 ▶).

Apoptosis plays a critical role in the regulation of normal cells homeostasis and cancer cells growth (Kuno et al., 2012 ▶). The present study was designed to shed light on the anti-cancer effects of Jujube on human breast and cervical cancer cells. We found that the Jujube extract decreased the cell viability, a response associated with increased Bax/Bcl-2 genes ratio.

## Materials and Methods

**Jujube aqueous extract preparation**

The semi-dried fruits of Jujube were washed and after seed removal, soft red parts were dried in 50°C and grounded into powder in a mortar. The powder was dissolved in boiling distilled water for 30minute, then filtered by a sterile filter (0.2 μm) and lyophilized (Hoshyar et al., 2015 ▶).

**Cell culture and Cell viability assay**

Cervical cancer cell line (OV2008) was kindly provided from Doctor Benjamin K. Tsang's laboratory (Department of Obstetrics, Gynecology and Cellular and Molecular Medicine, University of Ottawa, Canada). Breast cancer cell line (MCF-7) and normal cell line (MCF-10A) were purchased from Iranian Biological Resource Center, Iran. OV2008, MCF-7 and MCF-10A cells were cultured in RPMI and DMEM media, respectively (Abedini et al., 2004 ▶, Kobayashi et al., 2013 ▶). The cells were treated with different concentration of Jujube extract (0-3 mg/ml) for various interval times (0-72 hours). The stained cells by Trypan Blue observed and counted via an inverted microscope. MTT assay was used to assess the anti-proliferative effects of Jujube aqueous extract on the cancer cells (Wang et al., 2014 ▶). Using the dose- and time-dependent curves by linear interpolation, the IC50value of Jujube was calculated to analyze its cytotoxic efficiency (Bathaie et al., 2013 ▶).

**Quantitative Real Time-PCR analysis**

Total RNA was isolated using RNeasy Mini Kit (Qiagene-USA). The extracted RNA was immediately used in RT-PCR to generate first-strand cDNA (cDNA Synthesis Kit, Thermo Scientific, USA). The Quantitative RT-PCR for Bax and Bcl2was carried out using the specific primers (Table 1). Gene amplification was performed in the ABI Step One™ Real-Time PCR System (Applied Biosystems, Foster City, CA) with 40cycles of denaturation at 95°C for 30s, annealing and extension at 60°C for 30s and data collection 80°C for 20s. β-actin gene was used to normalize the relative expression for interested genes calculated by 2^ΔΔCT^ method and SYBR Green kit according to our previous reports (Abedini et al., 2014 ▶).

**Table 1:** Real-time primer sequences

**Gene**	**Sequences**
**β-Actin**	5'TGGCACCCAGCACAATGAA3' (Forward) 5' CTAAGTCATAGTCCGCCTAGAAGCA 3' (Reverse)
**Bax**	5' TGGAGCTGCAGAGGATGATTG3' (Forward) 5' GAAGTTGCCGTCAGAAAACATG3' (Reverse)
**Bcl2**	5'CTGCACCTGACGCCCTTCACC3' (Forward) 5'CACATGACCCCACCGAACTCAAAGA3' (Reverse**)**

**Statistical analysis**

Results are expressed as mean ± SEM for at least three independent experiments (n = 3). Data were analyzed using one-way ANOVA and with Tukey’s post hoc-test to assess differences between experimental groups (PRISM 5.0; Graph- Pad Software Inc.).

## Results

**Effects of Jujube on cancer cells proliferation**

**Figure 1:**
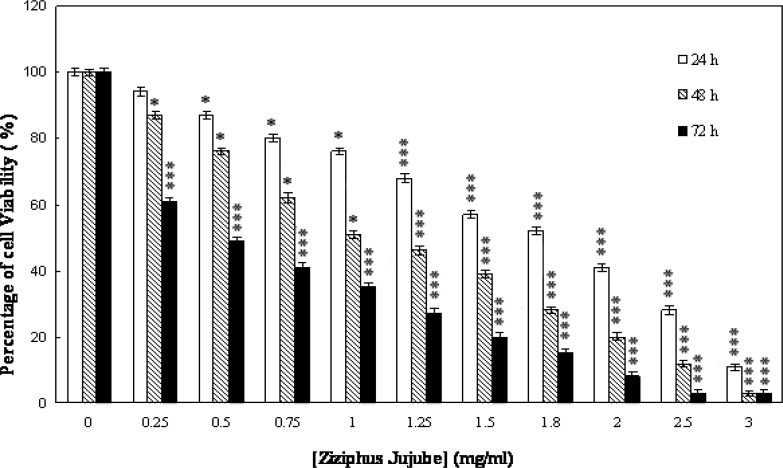
Effect of Jujubeon MCS-7 cell viability. The cells were treated with different concentrations of Jujube for 0-72 hours. Data are expressed as mean ± SEM (n=3). Values are statistically significant at ***p<0.001, *p<0.05 vs. respective control group (One-way ANOVA followed by Tukey’s post hoc test).

**Figure 2:**
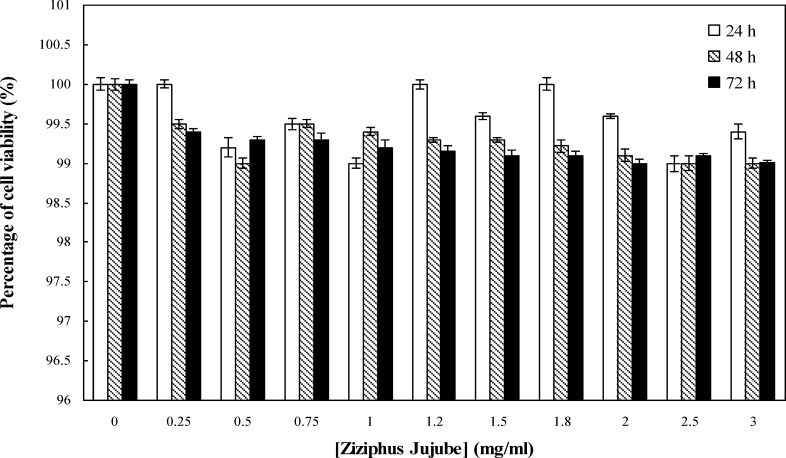
Effect of Jujubeon MCF-10A cell viability. The cells treated with different concentrations of Jujube for 0-72 hours. Results are reported as the mean ± SEM (n=3; p>0.05). Values are not statistically significant compared to the control group (One-way ANOVA followed by Tukey’s post hoc test

To examine the effect of Jujube on cell proliferation, cells were cultured and treated with Jujube extract (0-3 mg/ml; 0-72 hours). The effect of Jujube aqueous extract on the cancer cell morphology and proliferation were assessed by Trypan Blue staining and MTT assay, respectively. Jujube extract leads to cell shrinkage, blebbing and piknotic nuclei. In addition, Jujube treatments significantly decreased cell proliferation after 24 and 48 hours with 0.25-1 mg/ml (p<0.05) and 1.25-3 mg/ml h (p<0.001) as well as 72 hours with 0.25-3 mg/ml (p<0.001) in a dose- and time-dependent manner when compared with control group without Jujube (Figure 1). As shown in Table 2, the IC50 values of Jujube significantly decreased after different times (24-72 hours) in both cancer cell lines (p<0.01). This response was more evident in MCF-7 cells. In another word, IC50 of Jujube for MCF-7 after different times was higher compared to OV-2008 cells (P<0.05). Analyses of the cell survival showed that OV2008 cells were more sensitive to jujube compared to MCf-7 cells. Parallel treatment of the normal cells with this herb indicted a much less inhibitory effect on the viability of MCF-10A cells (Figure 2). Post hoc test revealed that Jujube at doses of 0.25-3 mg/ml (p=0.4487) did not affect growth of normal cells when compared with control group without Jujube.

**Table 2:** IC50 values (mg/ml) of Jujube in both cancer cells.

**Cancer cell line**	**24 hours**	**48 hours**	**72 hours**
**OV2008**	1.2 ± 0.03	0.5 ± 0.05a	0.2 ± 0.02a
**MCF-7**	1.8 ± 0.08b	1 ± 0.03a,b	0.5 ± 0.05a,b

Data are expressed as the means of triplication ± SEM (n=3). a p<0.01 IC50 for 48 and 72 hours in comparison with 24 hours treatment in both cell lines; b p<0.05 IC50 for MCF-7 cells in comparison with IC50 for OV 2008 cells at different time treatments

**Alteration of apoptosis regulating genes expression by Jujube in cancer cells**

**Figure 3:**
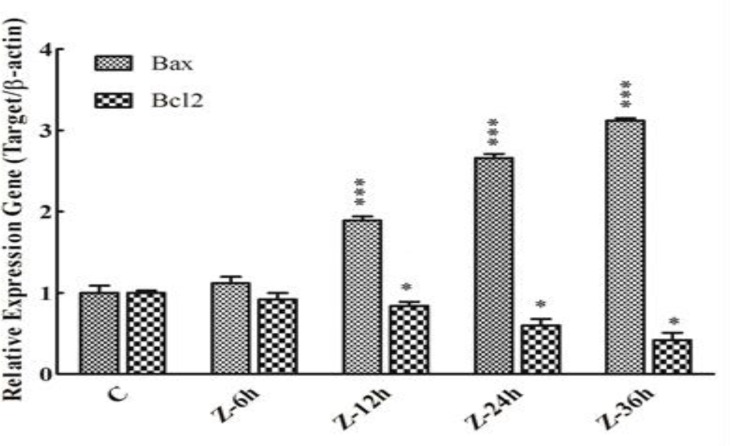
OV2008 cells treated with 1.2 mg/ml Jujube for 0-36h. Jujube increased the gene expression of Bax and decreased Bcl2 expression in cells. Data represents relative gene expression (Target/β-actin) mean ± SEM of three experiments (n=3). Values are statistically significant at ***p<0.001, *p <0.05 vs. respective control group (One-way ANOVA followed by Tukey’s post hoc test

Anti-proliferative effects of Jujube suggested that it may attenuate the cell proliferation through alteration of apoptotic regulating genes. To examine this hypothesis, we investigated Jujube’s effect on expression of Bax and Bcl2 genes. As indicated in Figure 3, herb significantly increased the expression of Bax (p<0.001) and decreased Bcl-2 expression (p<0.05) in cancer cells. Jujube also dramatically increased the Bax/Bcl-2 mRNA ratio as high as six folds in treated cancer cells.

## Discussion

Numerous studies have suggested that herbs exert potent anti-carcinogenic effects due to their ability to induce apoptosis. Some of these medicinal plants are *Crocus sativus* (Hoshyar et al., 2013 ▶), *Allium sativum* (Tsubura et al., 2011 ▶), *Camellia sinensis* (Thakur, 2012 ▶), *Aloevera *(Rajeswari et al., 2012 ▶), and *Curcuma longa *(Hashim et al., 2013 ▶). Jujubeis one of the most valuable herbs with terrific medicinal ingredients (Mahajan and Chopda, 2010 ▶). 

In the present study, we investigated the anti-proliferative effects of Jujube aqueous extract on MCF-7 and OV2008 cell lines which showed that it significantly inhibited cell growth in a dose- and time-dependent manner. We have also assessed the IC50 of Jujube and shown that its values decreased in a time-dependent manner on both cancer cells. Although there is a significant difference among different times (24-72 hours) for each cancer cell line, the IC50 measures of Jujube for OV2008 were markedly less than the values for MCF-7. This may suggest that OV2008 cells were more sensitive to effective dose of Jujube compared to MCF-7 cells. 

In this context, the anti-proliferative effect of de-proteinized polysaccharide (DPP) isolated from jujube on melanoma cells was evaluated and showed that IC50 of DPP was attenuated less than 20% between 24 and 48h treatment (Hung et al., 2012 ▶). However, in the present study, these decreases were 60 and 40% from 24h to 48h for OV2008 and MCF-7 cells, respectively. Moreover, the IC50 values of DPP in melanoma cells at different times were more than three folds in comparison with those of Jujube in OV2008 and MCF-7 (Hung et al., 2012 ▶). Additionally, it has been shown that cisplatin induces cell death at more extent in OV2008 compared to A2780s cells which are both chemosensitive cancer cells (Abedini et al., 2014 ▶). Plastina and coworkers indicted that the three alcoholic extraction of Jujube significantly inhibited proliferation and induced apoptosis in both estrogen receptor alpha (ERα) positive MCF-7 and ERα negative SKBR3 human breast cancer cells. However, they did not provide any result about their mechanism (Plastina et al., 2012 ▶). The present study is in line with their data. Our findings also illustrated that various concentrations of Jujube had no cytotoxic effect on MCF-10Anormal cells. Taken together, it seems that inhibitory effects of Jujube and/or its active metabolites on different cell lines are following the same trends, but illustrated various intensities. 

However, the precise mechanism of this response has not been reported yet. We recently have studied the impact of Jujube on gene expression which involves in the cell cycle regulation. We showed that it increased TP53, P27 and P21 mRNA abundance, a response which was associated with a decreased in CD1 mRNA level (Submitted manuscript under review). Moreover, we showed that aqueous Jujube alters the expression of apoptosis regulating genes including Bax and Bcl2 and their ratio. Dysregulation of apoptosis is associated with an imbalance of expression of genes which involvesin cell death and proliferation (Reed, 1999 ▶; Cheung et al., 2012 ▶). The Bcl-2 family genes consists of both pro-apoptotic and anti-apoptotic proteins e.g. Bax and Bcl-2, respectively (Elmore, 2007 ▶; Wong, 2011 ▶). The balance of their expression and distribution are key determinants for cell fate (Piltan et al., 2010 ▶). Bcl-2 located in the membrane of the nucleus and mitochondria and as a pro-survival molecule, sequesters and prevents Bax translocation to mitochondria resulting in apoptosis inhibition (Edlich and Banerjee, 2011 ▶). To further examine the mechanism(s) by which Jujube exerts the cell proliferation, here we evaluated its effects on apoptotic gene expression level. To our knowledge, this is the first quantitative assessment demonstrated that cell treatment with Jujube (1.2 mg/ml; 0-36h) resulted in more than three-fold increase in Bax mRNA level, a response associated with 50% decrease in Bcl-2 mRNA abundance, thereby significantly enhancing the Bax / Bcl-2mRNA ratio as much as 6 folds in treated cells. 

In summary these findings support the notion that Jujube exerts selective anti-tumor effect via inhibition of cell growth and induction of apoptosis. It could be apromising strategy to develop a successful treatment for cancer therapy.
